# Trajectories of autistic social traits in childhood and adolescence and disordered eating behaviours at age 14 years: A UK general population cohort study

**DOI:** 10.1111/jcpp.13255

**Published:** 2020-05-03

**Authors:** Francesca Solmi, Francesca Bentivegna, Helen Bould, William Mandy, Radha Kothari, Dheeraj Rai, David Skuse, Glyn Lewis

**Affiliations:** ^1^ Division of Psychiatry University College London London UK; ^2^ Centre for Academic Mental Health Population Health Science University of Bristol Bristol UK; ^3^ Gloucestershire Health and Care NHS Foundation Trust Gloucester UK; ^4^ Division of Psychology and Language Sciences University College London London UK; ^5^ Great Ormond Street Institute of Child Health University College London London UK; ^6^ NIHR Biomedical Research Centre University of Bristol Bristol UK; ^7^ Avon and Wiltshire Partnership NHS Mental Health Trust Bristol UK

**Keywords:** eating disorders, autistic traits, cohort study, trajectories, ALSPAC

## Abstract

**Background:**

Some people with eating disorders have difficulties with social communication. However, no longitudinal evidence regarding the direction of this association exists. We investigated trajectories of autistic social traits across childhood and adolescence in adolescents with and without disordered eating behaviours in early adolescence.

**Methods:**

We used data from the Avon Longitudinal Study of Parents and Children. Our disordered eating measure indicated presence of any, monthly and weekly disordered eating (fasting, purging, dieting, binge eating) at age 14 years. Autistic social traits were reported by mothers using the Social and Communication Disorders Checklist (SCDC) at age seven, 11, 14 and 16 years. We modelled SCDC score trajectories using multilevel negative binomial models adjusting for a number of child‐ and maternal‐level confounders.

**Results:**

Of the 5,381 adolescents included in our sample, 421 (7.8%) experienced one or more disordered eating behaviours, and 148 (2.8%) weekly episodes. Adolescents with disordered eating had a 20% increase in SCDC scores (relative risk (RR) 1.23, 95% confidence interval (CI):1.14, 1.32) compared to those without disordered eating. This association was particularly apparent for those reporting weekly (RR 1.43, 95%CI: 1.27, 1.61) as opposed to monthly disordered eating (RR 1.12, 95%CI: 1.01, 1.22).

**Conclusions:**

Greater autistic social traits in childhood could represent a risk factor for the development of disordered eating in adolescence. Although mechanisms of this association need to be elucidated, clinicians should be aware that autistic social traits could have predated the eating disorder when managing people with these conditions.

## Introduction

Clinicians have long been aware that some people with eating disorders present with social and flexibility difficulties that appear autistic in nature, suggesting that they might be on the autism spectrum, or have elevated levels of autistic traits (Gillberg, 1983). The hypothesis that autistic traits might be a risk factor for the development of eating disorders was first tested in the landmark Gothenburg Studies (Gillberg & Råstam, 1992; Nilsson et al., 1999; Rastam, 1992). This cohort followed 102 individuals (51 diagnosed with adolescent‐onset anorexia nervosa and 51 general population controls) for 20 years, assessing them for autism on four occasions. Researchers found that around 20% of those with anorexia nervosa also had autism and that these participants had poorer outcomes at follow‐up (Råstam, Gillberg, & Wentz, 2003).

These findings have been since replicated (Westwood et al., 2016; Westwood & Tchanturia, 2017). Using more rigorous assessment methods in different clinical populations with eating disorders, the prevalence of autism in adult samples has been consistently found to range between 20% and 30% (Vagni et al., 2016; Westwood et al., 2016; Westwood & Tchanturia, 2017). A number of investigations in general population samples also found an increased risk of comorbid autism spectrum disorder (ASD) in individuals diagnosed with anorexia nervosa and their relatives, which is nevertheless also observed for other conditions (Koch et al., 2015). In children and young people with eating disorders, the prevalence of autistic traits and diagnosis is lower than that observed in adults, ranging from 3% to 10% (Pooni et al., 2012; Rhind et al., 2014; Westwood et al., 2018), depending on the definition and type of assessment used.

Studies have also shown autistic traits can be comorbid with a range of disordered eating behaviours including binge eating, which is more typical of bulimia nervosa and binge eating disorders (Christensen et al., 2019), suggesting that autistic traits could represent a risk factor operating across the whole spectrum of eating disorder psychopathology. However, the largely cross‐sectional or case–control nature of these studies (Christensen et al., 2019; Vagni et al., 2016; Westwood et al., 2016; Westwood & Tchanturia, 2017) allows for the possibility that observed associations might be due to reverse causation. There is some evidence that the psychological effects of starvation can mimic the symptoms of autism (Hiller & Pellicano, 2013), and studies have shown that some theory of mind difficulties observed in anorexia nervosa abate as body weight is restored in recovery (Oldershaw et al., 2010).

We are not aware of any longitudinal general population studies that have measured autistic traits prior to the onset of eating disorder psychopathology. These are needed to assess the temporality of this association, a pre‐requisite for causal inference. Existing general population studies, as well as relying on cross‐sectional designs, also have a number of other limitations. Most employ small convenience samples, increasing the likelihood of type I or II error and selection bias, and limiting generalizability. These studies at times do not include a control group, and in many cases, only consider a subset of disordered eating behaviours (Christensen et al., 2019).

The aim of this study was to compare trajectories of autistic social traits using the Social and Communication Disorders Checklist (SCDC) from childhood to mid‐adolescence between adolescents with no disordered eating at age 14 years and those with any, monthly or weekly disordered eating behaviours at that same age. The SCDC is a measure of social communication problems, a core feature of ASD. Disordered eating behaviours – including dieting, fasting, binge eating and purging – occur across the whole eating disorder spectrum and are a risk factor for the development of eating disorders (Stice, 2002).

The advantage of this trajectory approach, which has been previously used in longitudinal research to better understand issues of reverse causation (Singh‐Manoux et al., 2017), is that it allowed us to simultaneously explore different hypothetical scenarios, which we show in Figure S1. If autistic traits were not a risk factor for disordered eating, we would find the two trajectories overlapping (Figure S1a). If the association between autistic traits and disordered eating were due to reverse causation (i.e. disordered eating results in increased autistic traits), trajectories of autistic social traits would start from similar values at age seven years in participants with and without disordered eating at age 14 years and would begin to diverge in early adolescence, when disordered eating is most likely to emerge (Figure S1b). If autistic traits preceded disordered eating (i.e. were thus a potential risk factor), we would find that the SCDC trajectories were already divergent between the different disordered eating groups in early childhood (Figure S1c). Finally, modelling trajectories until age 16 years, that is after our index measurement of disordered eating, allowed us to investigate whether trajectories of autistic traits become more divergent in mid‐adolescence, which would suggest that disordered eating might also affect the expression of autistic traits (Figure S1d).

## Methods

### Sample

The Avon Longitudinal Study of Parents and Children (ALSPAC) is a general population longitudinal birth cohort of children born in the former county of Avon (Bristol, UK). A total of 14,541 pregnancies with an expected delivery date falling between 1 April 1991 and 31 December 1992 were recruited in the study. Of the 14,062 live births, 13,988 children were alive at one year of age (Boyd et al., 2013; Fraser et al., 2013).

In this study, we included children who were part of the core ALSPAC sample and were alive at one year of age, and who had complete data on disordered eating behaviours at age 14 years and at least one SCDC measurement between the ages of seven and 16 years. This, coupled with imputation of missing data (see ‘Data analysis’ section), helped us minimizing the number of children lost to follow up and reduce the potential for selection bias. In line with previous research, for each set of twins (*n* = 179 pairs in our sample), we excluded one at random to avoid over‐/underestimation due to shared genetic and environmental effects (Bruckauf & Chzhen, 2016; Russell et al., 2019).

The ALSPAC Law and Ethics Committee and the Local Research Ethics committees gave ethical approval for the study. More details on the ALSPAC cohort can be found on the study website (www.bristol.ac.uk/alspac), which also contains details of all the data that are available through a fully searchable data dictionary available at http://www.bris.ac.uk/alspac/researchers/data‐access/data‐dictionary/.

### Disordered eating behaviours at age 14 years

At approximately 14 years of age, adolescents self‐reported the presence of dieting, fasting and purging for weight loss, and binge eating in the previous 12 months via postal questionnaires. These questions have been used in a number of previous studies as they capture core eating disorder behaviours (Bould et al., 2018; Micali et al., 2017).

Adolescents were asked the frequency with which they fasted (i.e. not eating for 24 hr with to losing weight) or purged (i.e. self‐induced vomiting, or use of laxatives/diet pills to lose weight) in the previous 12 months. Possible answers ranged from less than monthly to every day. We considered these behaviours present if participants reported them as occurring at least monthly and up to daily. Presence of binge eating was assessed with two questions. First, adolescents were asked how often they had eaten large amounts of food in a short period of time (i.e. 2 hr). Possible answers ranged from less than monthly to daily. Adolescents who reported any overeating were directed to a follow‐up question, asking whether, during these episodes, they felt out of control (i.e. they could not stop eating even if they wanted to). Possible answers were as follows: never, sometimes or frequently. We defined adolescents as binge eating if they reported at least monthly episodes of overeating and if they experienced loss of control ‘sometimes’ or ‘frequently’. For these three behaviours (fasting, bingeing and purging), we also created categorical variables indicating whether they never occurred; occurred monthly, but less than weekly; or occurred at least weekly.

Dieting was measured using two questions. Participants were asked whether they had dieted in the previous year and, if so, for how long. Adolescents were considered to be dieting if they reported dieting ‘several times’, ‘often’ or being ‘always’ on a diet and said that they dieted for at least 1 month and up to 12 months continuously.

From these individual variables, we created an overall binary indicator of whether the adolescent did or did not report any disordered eating behaviours (i.e. any binge eating, purging, or fasting occurring at least monthly, or dieting for a month or more). To test the presence of a dose–response association with more severe disordered eating presentations, we also created a three‐level categorical variable. This indicated whether the adolescents (a) did not have any disordered eating behaviours; (b) experienced episodes of fasting and/or purging and/or binge eating at least monthly (but less than weekly); (c) experienced weekly (and up to daily) episodes of fasting, purging or binge eating. Weekly episodes of binge eating and purging or fasting are diagnostic criteria for bulimia nervosa and binge eating disorder in DSM‐5; hence, we modelled our severity definition around these. Adolescents who only reported dieting were included in the monthly disordered eating category. If they reported other behaviours, they were included in the category corresponding to the frequency of the latter, that is monthly or weekly (see Figure S2).

### Autistic social traits

Parents reported their children’s autistic social traits using the Social and Communication Disorders Checklist (SCDC), when their study child was approximately seven, 11, 14 and 16 years of age. The SCDC is a measure of autistic social reciprocity and social communication difficulties in clinical settings and the general population (Skuse, Mandy, & Scourfield, 2005). The SCDC has strong internal consistency (α=.93) and test–retest stability (*r* = .81 over 2.7 years; Skuse, Mandy, & Scourfield, 2005) and has been extensively used in the existing literature to measure autistic social traits (Kothari et al., 2013; Pickard et al., 2017; Robinson et al., 2016; Skuse, Mandy, & Scourfield, 2005).

### Confounders

We adjusted our main analyses for a number of potential confounders of the association between autistic social traits and disordered eating (i.e. variables which the literature suggests could cause both, for rationale see Appendix S1). These were child’s sex, maternal highest level of education (compulsory, i.e. up to General Certificate of Secondary Education obtained at age 16 years/noncompulsory, that is A‐levels, obtained at age 18 years and above) and maternal lifetime history of eating disorders (none/anorexia nervosa/bulimia nervosa/both). We also adjusted for maternal age at delivery, depressive symptoms in pregnancy and pre‐pregnancy body mass index (BMI) – all used as continuous variables. At 12 weeks of gestation, mothers self‐reported their pre‐pregnancy height and weight, from which we derived BMI values. At 32 weeks of gestation, mothers also completed the Edinburgh Postnatal Depression Scale (EPDS). The EPDS is a 10‐item self‐report measure of depressive symptoms, whose scores range from zero to 30 with higher scores indicating greater depressive symptoms (Cox, Holden, & Sagovsky, 1987). The EPDS has been used to measure maternal depression in general population studies (Pearson et al., 2013; Ramchandani et al., 2005). We additionally adjusted for child’s BMI as a time‐varying confounder measured at time of assessment of each SCDC measurement (i.e. age seven, 11, 14 and 16 years).

Additional sensitivity analyses (see Data analysis section) were also adjusted for depressive symptoms at age 14 years measured with the short Moods and Feelings Questionnaire (Angold et al., 1995).

### Data analysis

We described our sample, overall and in relation to autistic social traits, using means with standard deviations and frequencies with proportions.

We modelled trajectories of SCDC scores in children with and without disordered eating behaviours at age 14 years using growth curve multilevel models, using repeated observations within each child. We used negative binomial models as opposed to linear models given the count nature of our variable and presence of overdispersion. As recommended in the literature (Rabe‐Hesketh & Skrondal, 2012), in each model, we included a continuous indicator of time (i.e. ages seven to 16 years, mean‐centred) and time squared in order to investigate the overall shape of the trajectories. We ran five models on the whole sample and by child’s sex, as there is evidence that trajectories of autistic social traits differ between boys and girls in this sample (Mandy et al., 2018).

For each disordered eating definition (i.e.: never/at least monthly; never/monthly/weekly) first, we ran an unconditional model including an indicator of time (mean‐centred) and time squared (Model 1). In Model 2, we included the disordered eating indicator, and in Model 3, we added all of our pre‐specified confounders. To test for the presence of differential associations by BMI, we included an interaction term between the disordered eating variable and BMI. We also investigated interactions with time and time squared in two separate models (models 4 and 5). This was to test whether the rate of change in (i.e. the shape of) SCDC score trajectories differed between participants with and without disordered eating and explore the different hypotheses we set out to investigate (see Introduction). As fewer males than females reported disordered eating behaviours, we did not have had enough statistical power to formally test for the presence of an interaction with time using the none, monthly and weekly disordered eating indicators in males. Hence, for this group, we only used the no/any disordered eating indicator in sex‐stratified analyses.

We imputed missing confounder data as well as outcome data for children with one to three missing observations (proportions shown in the ‘Results’ section), using multiple imputation with chained equations imputing 50 datasets. More details are provided in Appendix S2.

We present, as main analyses, those run on children with at least one SCDC measurement, complete disordered eating data and imputed missing confounder data. In supplemental material, we present results of our main analyses based on: adolescents with at least two SCDC measurements, complete disordered eating data and imputed missing confounder data; and adolescents with at least one SCDC measurement and imputed missing disordered eating and confounder data. We also ran the analyses using multilevel mixed linear models with random effects of time, based on the sample of adolescents with at least one SCDC measurement, complete data on disordered eating and imputed confounders.

As additional sensitivity analyses, we ran a univariable and multivariable logistic regression model testing the association between autistic social traits at age seven years and disordered eating at 14 years. In these analyses, we adjusted for all confounders included in the trajectories’ models, with the exception of time. We also ran a univariable and multivariable negative binomial regression model testing the association between disordered eating at age 14 years and autistic social traits at age 16 years. In these models, in addition to all previously specified confounders, we also adjusted our models for autistic traits and depressive symptoms at baseline (i.e. age 14 years), as we hypothesized these could confound the association under study.

We conducted all analyses in Stata 15 (StataCorp, 2017).

## Results

### Sample and missing data

Of the children in the core ALSPAC sample (*N* = 14,062), 13,793 (98.1%) were alive at one year and, of these, 9,185 (66.6%) had at least one SCDC measurement between ages seven and 16 years. Compared with children without any SCDC measurements, a greater proportion of those with at least one measurement available had older mothers, who had completed noncompulsory education and who had lower levels of depression (data available from authors).

Among children who had data available on disordered eating at age 14 years (*n* = 5,614), 5,381 (93.8%, 2,410 boys and 2,971 girls) had, at least, one SCDC measurement available and were thus included in our models (see flowchart of participation in Figure S3). Of these, 3,301 (61.4%) children had SCDC data available at all ages assessed, 1,192 (22.2%) at three time points, 555 (10.3%) at two time points and 333 (6.2%) at one time point.

Children with missing disordered eating data had mothers who were younger, were less educated, had greater depressive symptoms and had higher pre‐pregnancy BMI. A greater proportion of boys and children with greater SCDC scores had missing information on disordered eating at 14 years (see Table S1).

The majority of children in our sample had a mother who only completed compulsory education (53.7%), was 26–35 years of age at their birth (71.2%), had a normal pre‐pregnancy BMI (76.7%) and did not have high depressive symptoms (89.7%) or a history of eating disorders (96.5%).

Overall, SCDC scores decreased in the sample between age seven and 11 years, but then increased again at 14 and 16 years (Table 1 and Table S2). Boys had greater SCDC scores, and so did children whose mothers were younger and had an underweight or obese pre‐pregnancy BMI, or greater depressive symptoms in pregnancy (Table 1).

**Table 1:** Sample characteristics (sample based on children with at least one Social and Communication Disorders Checklist measurements and complete disordered eating data, N = 5,381)

	*N* (%)	Social and Communication Disorders Checklist			
		7 years Mean (*SD*)	11 years Mean (*SD*)	14 years Mean (*SD*)	16 years Mean (*SD*)
Mean SCDC scores		2.5 (3.3)	2.1 (3.1)	2.3 (3.3)	2.6 (3.6)
Mean participants’ age (in years)		7.6 (0.12)	10.7 (0.13)	13.9 (0.15)	16.8 (0.4)
Participants’ age range (in years)		7.5–9.0	10.6–14.8	13.7–16.1	16.6–18.1
Child’s sex					
Male	2,410 (44.8%)	2.9 (3.7)	2.3 (3.5)	2.4 (3.6)	2.6 (3.7)
Female	2,971 (55.2%)	2.3 (3.0)	1.8 (2.8)	2.2 (3.0)	2.7 (3.6)
Child’s ethnicity					
White	4,992 (96.4%)	2.6 (3.4)	2.0 (3.1)	2.3 (3.3)	2.6 (3.6)
Ethnic minority	189 (3.6%)	2.6 (3.3)	3.4 (3.4)	2.5 (3.8)	3.2 (3.8)
Maternal Education					
Compulsory	2,819 (53.7%)	2.6 (3.4)	2.1 (3.2)	2.3 (3.3)	2.7 (3.7)
Noncompulsory	2,432 (46.3%)	2.5 (3.3)	2.0 (3.1)	2.3 (3.3)	2.6 (3.5)
Maternal eating disorder					
None	5,080 (96.5%)	2.6 (3.3)	2.1 (3.1)	2.3 (3.3)	2.6 (3.7)
Anorexia nervosa or Bulimia nervosa	182 (3.5%)	2.3 (2.7)	1.9 (2.9)	2.1 (3.0)	2.5 (3.2)
Maternal age					
15–19	81 (1.5%)	3.5 (4.3)	2.7 (3.6)	2.4 (3.6)	3.3 (3.8)
20–25	989 (18.4%)	2.6 (3.3)	2.1 (3.2)	2.4 (3.8)	2.7 (3.6)
26–35	3,834 (71.2%)	2.5 (3.3)	2.0 (3.1)	2.3 (3.3)	2.6 (3.6)
36–44	477 (8.9%)	2.7 (3.2)	2.0 (3.2)	2.0 (2.9)	2.7 (3.7)
Maternal depressive symptoms					
No	4,438 (89.7)	2.5 (3.2)	1.9 (3.0)	2.2 (3.2)	2.5 (3.5)
Yes	511 (10.3)	3.4 (3.9)	2.8 (3.6)	3.0 (3.8)	3.4 (4.3)
Maternal Pre‐pregnancy BMI					
Underweight	220 (4.4%)	2.9 (3.6)	2.5 (3.4)	2.6 (3.3)	2.7 (3.4)
Normal weight	3,804 (76.7%)	2.5 (3.2)	2.0 (3.1)	2.2 (3.2)	2.7 (3.7)
Overweight	690 (13.9%)	2.6 (3.4)	2.2 (3.3)	2.4 (3.5)	2.5 (3.6)
Obese	244 (5.0%)	2.7 (3.5)	2.0 (2.7)	2.4 (3.4)	2.7 (3.3)

BMI, body mass index; SCDC, Social and Communication Disorders Checklist.

### Disordered eating

At age 14 years, 334 (11.2%) girls reported at least one disordered eating behaviour, with 218 (7.3%) experiencing these behaviours monthly and 116 (3.9%) weekly. Among boys, these behaviours were less common. A total of 87 (3.6%) boys said they had had episodes of disordered eating in the previous year, with 55 (2.3%) reporting monthly and 32 (1.3%) weekly episodes. Prevalence of individual behaviours is presented in Table S3.

### Trajectories of autistic social traits in children with disordered eating

In Table S2, we show results of unconditional models (Model 1).

As can be seen in Table 2, in both univariable and multivariable models, adolescents who had disordered eating behaviours at 14 years of age had overall greater SCDC scores (univariable model 2 relative risk [RR]: 1.25, 95% confidence interval [CI]: 1.16, 1.34, *p* < .0001; multivariable model 3 RR: 1.23,95% CI: 1.14, 1.32) compared with those who did not. This difference was greater among adolescents with weekly disordered eating behaviours (univariable model 2 RR: 1.45, 95%CI: 1.29, 1.63; multivariable model 3 RR: 1.43; 95%CI: 1.27, 1.62) than in those with monthly behaviours (univariable model 2 RR: 1.13, 95%CI: 1.04, 1.24; multivariable model 3 RR: 1.12; 95%CI: 1.01, 1.22). There was no evidence of an interaction between any disordered eating and BMI (*p* = .4569) and monthly/weekly disordered eating and BMI (*p* = .7860, data available from authors).

**Table 2:** Multilevel negative binomial regression modelling trajectories of social communication difficulties between age 7 and 16 years among participants with disordered eating at age 14 years. Sample based on participants with complete disordered eating data, at least one SCDC measurement and imputed confounders. (*N* = 5,381)

Autistic social traits				
	Univariable model 2 Relative risk^a^ (95% CI)	Adjusted model 3 Relative risk^a^ (95% CI)	Adjusted model 4 Relative risk^a^ (95% CI)	Adjusted model 5 Relative risk^a^ (95% CI)
Any disordered eating behaviours				
No	Reference	Reference	Reference	Reference
Yes	1.25 (1.16, 1.34), *p* < .0001	1.23 (1.14, 1.32), *p* < .0001	1.22 (1.14, 1.32), *p* < .0001	1.22 (1.09, 1.38), *p* = .0008
Any DEB × time		–	1.02 (0.99, 1.04), *p* = .11	1.02 (0.99, 1.04), *p* = .15
Any DEB × time^2^		–	–	0.99 (0.99, 1.01), *p* = .97

	Univariable model 2 Relative risk^a^ (95% CI)	Adjusted model 3 Relative risk^a^ (95% CI)	Adjusted model 4 Relative risk^a^ (95% CI)	Adjusted model 5 Relative risk^a^ (95% CI)
Severity of disordered eating behaviours^a^				
None		Reference	Reference	Reference
Behaviours occurring monthly	1.13 (1.04, 1.24), *p* = .005	1.12 (1.01, 1.22), *p* = .01	1.12 (1.02, 1.22), *p* = .02	1.09 (0.93, 1.26), *p* = .28
Behaviours occurring weekly	1.45 (1.29, 1.63), *p* < .0001	1.43 (1.27, 1.62), *p* < .0001	1.43 (1.27, 1.60), *p* < .0001	1.49 (1.23, 1.80), *p* < .0001
Monthly DEB × time		–	1.02 (0.99, 1.04), *p* = .19	1.02 (0.99, 1.05), *p* = .16
Weekly DEB × time		–	1.02 (0.98, 1.05), *p* = .329	1.01 (0.98, 1.05), *p* = .50
Monthly DEB × time^2^		–	–	1.002 (0.99, 1.01), *p* = .63
Weekly DEB × time^2^		–	–	0.997 (0.99, 1.01), *p* = .57

Model 1 is presented in supplemental material and only contains the two time variables (i.e. time and time^2^).

Model 2 = Model 1 + disordered eating variable.

Model 3 = Model 2 + child’s sex, maternal age, maternal pre‐pregnancy BMI, maternal depressive symptoms in pregnancy, maternal history of eating disorders and maternal education. Child BMI was adjusted for as a time‐varying confounder.

Model 4 = Model 3 + eating disorder variable*time interaction

Model 5 = Model 4 + eating disorder variable* time^2^ interaction

Abbreviations: BMI, body mass index; CI, confidence interval; DEB, disordered eating behaviour; SCDC, Social and Communication Disorders Checklist.

^a^ Relative risk is derived from exponentiating the coefficients of multilevel negative binomial regressions, modelling the logs of SCDC scores in adolescents with disordered eating (i.e. any, monthly or weekly) compared to those with no disordered eating, holding confounder variables in the model constant.

**Figure 1:**
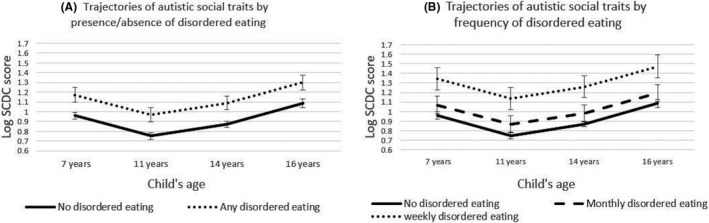
Trajectories of autistic social traits in children reporting at least one disordered eating behaviour (Panel A) and in those reporting monthly and weekly disordered eating behaviour (Panel B) at age 14 years, derived from Model 3 predictions. Sample based on participants with complete outcome data, at least one SCDC measurement and imputed confounders. (*N* = 5,381). Abbreviation: SCDC, Social and Communication Disorders Checklist

We found no evidence of any interactions (Table 2) with time or time squared terms. This means that in children with weekly disordered eating, autistic social traits were consistently higher across childhood and adolescence with no differences in rates of growth of these problems. This is illustrated in Figure 1.

### Trajectories of autistic social traits in boys and girls with disordered eating

In Table S4, we show results of unconditional models (Model 1) stratified by participants’ sex. In line with previous research (Mandy et al., 2018), we found that, overall, trajectories of autistic social traits differed by child’s sex. However, the association between disordered eating and trajectories of SCDC scores was similar in both groups. In univariable and multivariable models, both girls (univariable model 2 RR: 1.27, 95%CI: 1.17, 1.38; multivariable model 3 RR: 1.21, 95% CI: 1.12, 1.32) and boys (univariable model 2 RR: 1.37, 95%CI: 1.18, 1.60; multivariable model 3 RR: 1.30, 95%CI: 1.12, 1.52) with any disordered eating had consistently higher SCDC scores than comparison children, with no evidence of interactions with time (Table S5 and Figure S4).

Among girls, in both univariable and multivariable models, those with weekly disordered eating behaviours (univariable model 2 RR: 1.56, 95%CI: 1.37, 1.78; multivariable model 3 RR: 1.48, 95%CI: 1.30, 1.68) had greater SCDC scores than those with no disordered eating behaviours (Table S6 and Figure S5). Those with monthly disordered eating behaviours had greater SCDC scores than those with no disordered eating in the univariable model (Model 2 RR: 1.11, 95%CI: 1.01, 1.23), but this association became weaker in the multivariable model (Model 3 RR: 1.07, 95%CI: 0.97, 1.19). We also found no evidence of interactions with time (Table S6).

### Association between autistic traits at age seven years and disordered eating at age 14 years

As shown in Table 3, in the univariable model, a one standard deviation increase in SCDC scores at age seven years was associated with greater odds of any disordered eating (odds ratio (OR): 1.12, 95%CI: 1.01, 1.24, *p* = .02) and greater risk of weekly (relative risk ratio [RRR]: 1.19, 95%CI: 1.03, 1.38, *p* = .02) disordered eating at age 14 years.

**Table 3:** Univariable and multivariable logistic (any disordered eating) and multinomial logistic (frequency of disordered eating) regression models testing the association between autistic social traits (SCDC scores) at age 7 years and disordered eating at 14 years. Sample based on children with complete exposure and imputed outcome and confounders (*n* = 7,794)

	Any disordered eating at 14 years		Frequency of disordered eating at 14 years			
	Any Univariable model OR	Any Multivariable model OR	Monthly Univariable model RRR	Weekly Univariable model RRR	Monthly Multivariable model RRR	Weekly Multivariable model RRR
SCDC scores age 7 years	1.12 (1.01, 1.24), *p* = .02	1.18 (1.06, 1.32), *p* = .004	1.08 (0.95, 1.23), *p* = .22	1.19 (1.03, 1.38), *p* = .02	1.14 (1.00, 1.31), *p* = .06	1.24 (1.06, 1.46), *p* = .008

Multivariable models adjusted for child’s sex and BMI at age 7 years; maternal: education, age, prenatal BMI, history of eating disorders and depressive symptoms. OR, odds ratio; SCDC, Social and Communication Disorders Checklist; RRR, relative risk ratio.

In the multivariable model, higher SCDC scores at age seven years were still associated with greater odds of reporting any disordered eating at age 14 years (OR: 1.18, 95%CI: 1.06, 1.32, *p* = .004). After adjustment for confounders, there was also some evidence that a one standard deviation increase in SCDC scores was associated with an increased risk of monthly disordered eating behaviours (RRR: 1.14, 95%CI: 1.00, 1.31, *p* = .06) and good evidence of an increased risk of weekly behaviours (RRR: 1.24, 95%CI: 1.06, 1.46, *p* = .008).

### Association between disordered eating at age 14 years and autistic traits at age 16 years

As seen in Table 4, in univariable models, there was evidence that any (relative risk [RR]: 1.30, 95%CI: 1.13, 1.49, *p* = .0002), monthly (RR: 1.20, 95%CI: 1.01, 1.41, *p* = .04) and weekly (RR: 1.47, 95%CI: 1.17, 1.84, *p* = .0008) disordered eating behaviours were associated with greater SCDC scores at age 16 years. After adjustment for confounders, none of these associations were present (any disordered eating RR: 1.07, 95%CI: 0.94, 1.22, *p* = .28; monthly any disordered eating RR: 1.04, 0.89, 1.21, *p* = .66; weekly any disordered eating RR: 1.13, 95%CI: 0.92, 1.40, *p* = .23).

**Table 4:** Univariable and multivariable negative binomial regression model testing the association between any/monthly/weekly disordered eating behaviours at age 14 years and autistic social traits at age 16 years. Model based on participants with complete exposure data and imputed confounder and outcome data

	SCDC scores at age 16 years	
	Any univariable model RR^a^	Any multivariable model RR^a^
Any disordered eating at age 14 years		
Any vs. none	1.29 (1.13, 1.49) *p* = .0002	1.07 (0.95, 1.22), *p* = .28
Frequency of disordered eating at age 14 years		
Monthly vs. none	1.20 (1.01, 1.41), *p* = .04	1.04 (0.85, 1.21), *p* = .66
Weekly vs. none	1.47 (1.17, 1.84), *p* = .0008	1.14 (0.92, 1.40), *p* = .23

Multivariable model adjusted for child’s sex and BMI, depressive symptoms and autistic traits at age 14 years (baseline); maternal: education, age, prenatal BMI, history of eating disorders and depressive symptoms.

Abbreviations: RR, relative risk; SCDC, Social and Communication Disorders Checklist.

^a^ Relative risk is derived from exponentiating the coefficients of negative binomial regressions, modelling the logs of SCDC scores in adolescents with disordered eating (i.e. any, monthly or weekly) compared to those with no disordered eating, holding confounder variables in the model constant.

### Sensitivity analyses

Results of sensitivity analyses pertaining to trajectory modelling did not differ qualitatively from those of our main analyses (more details in Appendix S3 and Tables S7–S9).

## Discussion

In this study, we found that adolescents who experienced disordered eating behaviours at age 14 years had higher scores on the SCDC scale, measuring autistic social traits, throughout childhood and up to mid‐adolescence. By modelling trajectories of autistic social traits from childhood to mid‐adolescence, we were able to show that these differences in scores were already present (i.e. trajectories were already divergent) at age seven years when disordered eating behaviours are rare. This suggests that it is likely that autistic traits could constitute a risk factor for disordered eating (Figure S1c) as opposed to disordered eating leading to greater autistic social traits over time. This hypothesis was further supported by the results of our sensitivity analyses. Here, we found that while SCDC scores at seven were associated with disordered eating at 14 years, the latter were not associated with higher SCDC scores at 16 years after accounting for baseline values and confounders.

We also found that patterns of association were similar in girls and boys, although the low prevalence of disordered eating in the latter group meant we were unable to investigate associations by frequency of these behaviours. Since the majority of research to date has focused on girls, this finding is novel and warrants more research.

Finally, in both main and sensitivity analyses, we observed a dose–response association between greater SCDC scores and more frequent disordered eating behaviours, pointing to an association between greater difficulties with social communication in childhood and more severe disordered eating presentations.

### Limitations of this study

Our findings should be interpreted with some limitations in mind. Like any long‐term cohort study, the ALSPAC sample is characterized by high levels of attrition. However, we minimized the potential for selection bias by using multilevel models – which allowed us to include participants so long as they had at least one SCDC measurement available – and by imputing missing confounder data. Our findings were consistent across different sample specifications (see: Table 2 and Tables S5–S7).

Though cross‐sectional and case–control evidence on autistic traits and disordered eating exists (Christensen et al., 2019), to date, most research has focused on anorexia nervosa (Westwood & Tchanturia, 2017). We were not able to investigate associations between autistic social traits and specific eating disorder diagnoses. Eating disorder diagnoses in the community are relatively uncommon and difficult to derive from cohort data due to the complex nature of their presentation. For this reason, we measured disordered eating, which is more prevalent and captures behaviours that are also relevant to bulimia nervosa, binge eating disorder and atypical anorexia nervosa (i.e. anorexia nervosa without underweight BMI). People with these conditions are more likely to be missed in clinical settings; hence, general population studies are key in understanding their aetiology.

We cannot fully exclude the possibility that reverse causation could have occurred (i.e. that disordered eating was already present at age 7 years). However, we believe that this is unlikely as eating disorders such as anorexia nervosa, bulimia nervosa and binge eating disorder are virtually absent in this age group (Nicholls, Lynn, & Viner, 2011).

We adjusted our models for BMI based on the hypothesis that low BMI could result in symptoms typical of autism. However, since BMI was measured concomitantly to autistic social traits, we cannot fully exclude that BMI could have been a consequence of the latter. Nevertheless, the inclusion of BMI as a confounder did not appear to affect our estimates. Hence, we believe that it is unlikely that our results could be have been biased by the inclusion of a factor lying on the causal pathway between autistic social traits and disordered eating.

Compared to girls, a relatively small proportion of boys reported disordered eating behaviours at age 14 years, resulting in limited statistical power to test associations in this group. Recent evidence suggests that eating disorders might present differently in males. For instance, they might be more concerned with muscularity leading to different types of weight‐controlling behaviours (e.g. exercise to gain weight, use of steroids and other muscle enhancing products; Neumark‐Sztainer et al., 2014). While future studies should aim to capture these behaviours in order to expand eating disorder research to this group, we believe that our findings are important particularly in the light of the dearth of research on males with eating disorders.

### Comparison with previous studies, interpretation of findings and possible mechanisms

This is the first longitudinal study investigating the association between autistic social traits using the SCDC in childhood and adolescence, and disordered eating in adolescence. Our results are consistent with the findings of four cross‐sectional studies that investigated the association between disordered eating behaviours and autistic traits using the Autism‐Spectrum Quotient (AQ) scale (Baron‐Cohen et al., 2001; Christensen et al., 2019). Three studies reported specific associations between poor communication and social skills (as well as attention to detail) and disordered eating behaviours such as binge eating and vomiting, which constituted key behaviours in our disordered eating measure (Christensen et al., 2019).

Clinical accounts of women with eating disorders point to the presence of difficulties with social interactions and flexibility that predate the onset of their eating disorder in adolescence (Mandy & Tchanturia, 2015), and this is what we observed in this study. Previous research has shown that autistic traits in childhood are associated with increased anxiety and depression emerging as early as late childhood/early adolescence (Kanne, Christ, & Reiersen, 2009; Rai et al., 2018). One hypothesis is that difficulties in social communication, although already present at young ages, might become salient for the aetiology of eating disorders during adolescence via these increased depression or anxiety symptoms.

Adolescence is a time characterized by extensive changes in neurodevelopment and a period during which peer relationships become increasingly important and complex (Patton et al., 2016). Difficulties in communicating effectively during this period of social growth might result in problems with establishing and maintaining friendships and therefore difficult emotions, anxiety and low mood. Disordered eating, and perhaps other mental health difficulties with a primarily adolescent age of onset, might result from dysfunctional methods of coping with these emotional difficulties. Future studies should therefore test these hypotheses as possible mechanisms linking autistic social traits to disordered eating in order to devise appropriate preventative strategies.

It might be helpful for clinicians to consider social communication difficulties as a potential contributory factor when managing someone with an eating disorder. Our evidence suggests that autistic social traits can predate the onset of eating disorders and may not be solely a consequence of the illness. Establishing whether social communication difficulties preceded the onset of disordered eating behaviours in young people presenting with an eating disorder is likely to be challenging given the impact of starvation and low mood on some of their presentations, and diagnoses of autism spectrum conditions in this group should be made cautiously. However, young people with eating disorders may benefit from a greater understanding of their difficulties in this area and support in overcoming them.

## Supporting information

**Appendix S1.** Choice of confounders.
**Appendix S2.** Multiple imputation.
**Appendix S3.** Sensitivity analyses.
**Figure S1.** Graphical representation of expected trajectories under different hypothetical scenarios.
**Figure S2.** Definition of disordered eating behaviors in the disordered eating variable.
**Figure S3.** Flowchart of study participation.
**Figure S4.** Trajectories of autistic social traits by child’s sex and presence of disordered eating behaviours at age 14 years, derived from Model 3 predictions. Sample based on participants with complete disordered eating data, at least 1 SCDC measurements and imputed confounders. (*N* girls = 2,464, *N* boys = 1,890).
**Figure S5.** Trajectories of autistic social traits in girls by frequency of disordered eating behaviours at age 14 years, derived from Model 3 predictions. Sample based on participants with complete disordered eating data, at least 1 SCDC measurements and imputed confounders. (*N* = 2,464).
**Table S1.** Predictors of missing disordered eating data among girls with at least one SCDC measurements available.
**Table S2.** Results of the unconditional model (model 1) only modelling trajectories of autistic social traits between age 7 and 16 years among participants by age and age squared values. Sample based on participants with complete disordered eating data, at least one SCDC measurement and imputed confounders. (*N* = 5,381).
**Table S3.** Proportion of children with disordered eating behaviours in the main analytical sample, *n* = 5,381.
**Table S4.** Results of the unconditional model (model 1) only modelling trajectories of autistic social traits between age 7 and 16 years among participants by age and age squared values in boys and girls, separately. Sample based on participants with complete disordered eating data, at least one SCDC measurement and imputed confounders. (Girls, *n* = 2,971, boys, *n* = 2,410).
**Table S5.** Multilevel negative binomial regression modelling trajectories of social communication difficulties between age 7 and 16 years among participants with disordered eating at age 14 years stratified by participants’ sex. Sample based on participants with complete disordered eating data, at least one SCDC measurement and imputed confounders. (Girls, *n* = 2,971, boys, *n* = 2,410).
**Table S6.** Multilevel negative binomial regression modelling trajectories of social communication difficulties between age 7 and 16 years among girls with monthly and weekly disordered eating at age 14 years. Sample based on participants with complete disordered eating data, at least one SCDC measurement and imputed confounders. (*n* = 2,971).
**Table S7.** Multilevel negative binomial regression modelling trajectories of social communication difficulties between age 7 and 16 years among participants with disordered eating at age 14 years. Sample based on adolescents with complete disordered eating data, at least two SCDC measurements, and imputed confounders (*n* = 5,048).
**Table S8.** Multilevel negative binomial regression modelling trajectories of social communication difficulties between age 7 and 16 years among participants with disordered eating at age 14 years. Sample based on adolescents with at least one SCDC measurements, and imputed disordered eating and confounders (*n* = 9,185).
**Table S9.** Multilevel linear mixed regression modelling trajectories of social communication difficulties between age 7 and 16 years among participants with disordered eating at age 14 years. Sample based on adolescents with at least two SCDC measurements, complete disordered eating and imputed confounders. (*n* = 5,831).
