# Supplementary material for: Trajectories of autistic social traits in childhood and adolescence and disordered eating behaviours at age 14 years: A UK general population cohort study
Source: J Child Psychol Psychiatry. 2020 May 3;62(1):75–85. doi: 10.1111/jcpp.13255 (PMC8425328; doi:10.1111/jcpp.13255)
Supplement: Supplementary file 1 — Appendix S1. Choice of confounders. Appendix S2. Multiple imputation. Appendix S3. Sensitivity analyses. Figure S1. Graphical representation of expected trajectories under different hypothetical scenarios. Figure S2. Definition of disordered eating behaviors in the disordered eating variable. Figure S3. Flowchart of study participation. Figure S4. Trajectories of autistic social traits by child’s sex and presence of disordered eating behaviours at age 14 years, derived from Model 3 predictions. Sample based on participants with complete disordered eating data, at least 1 SCDC measurements and imputed confounders. (N girls = 2,464, N boys = 1,890). Figure S5. Trajectories of autistic social traits in girls by frequency of disordered eating behaviours at age 14 years, derived from Model 3 predictions. Sample based on participants with complete disordered eating data, at least 1 SCDC measurements and imputed confounders. (N = 2,464). Table S1. Predictors of missing disordered eating data among girls with at least one SCDC measurements available. Table S2. Results of the unconditional model (model 1) only modelling trajectories of autistic social traits between age 7 and 16 years among participants by age and age squared values. Sample based on participants with complete disordered eating data, at least one SCDC measurement and imputed confounders. (N = 5,381). Table S3. Proportion of children with disordered eating behaviours in the main analytical sample, n = 5,381. Table S4. Results of the unconditional model (model 1) only modelling trajectories of autistic social traits between age 7 and 16 years among participants by age and age squared values in boys and girls, separately. Sample based on participants with complete disordered eating data, at least one SCDC measurement and imputed confounders. (Girls, n = 2,971, boys, n = 2,410). Table S5. Multilevel negative binomial regression modelling trajectories of social communication difficulties between age 7 and 16 years [file JCPP-62-75-s001.docx]

**Supporting information – Trajectories of autistic social traits in childhood and adolescence and disordered eating behaviours at age 14 years. A UK general population cohort study – by Solmi *et al*.**

**Table of Contents**

[Figure S1: Graphical representation of expected trajectories under different hypothetical scenarios 3](#_Toc32226005)

[Figure S2: Definition of disordered eating behaviors in the disordered eating variable 4](#_Toc32226006)

[Appendix S1: Choice of confounders 4](#_Toc32226007)

[Appendix S2: Multiple imputation 5](#_Toc32226008)

[Figure S3: Flowchart of study participation 6](#_Toc32226009)

[Table S1: Predictors of missing disordered eating data among girls with at least one SCDC measurements available 7](#_Toc32226010)

[Table S2: Results of the unconditional model (model 1) only modelling trajectories of autistic social traits between age 7 and 16 years among participants by age and age squared values. Sample based on participants with complete disordered eating data, at least one SCDC measurement and imputed confounders. (N = 5,381) 8](#_Toc32226011)

[Table S3: Proportion of children with disordered eating behaviours in the main analytical sample , n=5,381 9](#_Toc32226012)

[Table S4 Results of the unconditional model (model 1) only modelling trajectories of autistic social traits between age 7 and 16 years among participants by age and age squared values in boys and girls, separately. Sample based on participants with complete disordered eating data, at least one SCDC measurement and imputed confounders. (Girls, n= 2,971, boys, n= 2,410) 10](#_Toc32226013)

[Figure S4: Trajectories of autistic social traits by child’s sex and presence of disordered eating behaviours at age 14 years, derived from Model 3 predictions. Sample based on participants with complete disordered eating data, at least 1 SCDC measurements and imputed confounders. (N girls = 2,464, N boys = 1,890) 11](#_Toc32226014)

[Figure S5: Trajectories of autistic social traits in girls by frequency of disordered eating behaviours at age 14 years, derived from Model 3 predictions. Sample based on participants with complete disordered eating data, at least 1 SCDC measurements and imputed confounders. (N = 2,464) 12](#_Toc32226015)

[Table S5: Multilevel negative binomial regression modelling trajectories of social communication difficulties between age 7 and 16 years among participants with disordered eating at age 14 years stratified by participants’ sex. Sample based on participants with complete disordered eating data, at least one SCDC measurement and imputed confounders. (Girls, n= 2,971, boys, n= 2,410) 13](#_Toc32226016)

[Table S6: Multilevel negative binomial regression modelling trajectories of social communication difficulties between age 7 and 16 years among girls with monthly and weekly disordered eating at age 14 years. Sample based on participants with complete disordered eating data, at least one SCDC measurement and imputed confounders. (n = 2,971) 14](#_Toc32226017)

[Appendix S3: Sensitivity analyses 15](#_Toc32226018)

[Table S7: Multilevel negative binomial regression modelling trajectories of social communication difficulties between age 7 and 16 years among participants with disordered eating at age 14 years. Sample based on adolescents with complete disordered eating data, at least two SCDC measurements, and imputed confounders (n=5,048). 16](#_Toc32226019)

[Table S8: Multilevel negative binomial regression modelling trajectories of social communication difficulties between age 7 and 16 years among participants with disordered eating at age 14 years. Sample based on adolescents with at least one SCDC measurements, and imputed disordered eating and confounders (n=9,185). 17](#_Toc32226020)

[Table S9: Multilevel linear mixed regression modelling trajectories of social communication difficulties between age 7 and 16 years among participants with disordered eating at age 14 years. Sample based on adolescents with at least two SCDC measurements, complete disordered eating and imputed confounders. (n=5,831) 18](#_Toc32226021)

[References 19](#_Toc32226022)

# Supplemental Figure 1: graphical representation of expected trajectories under different hypothetical scenarios


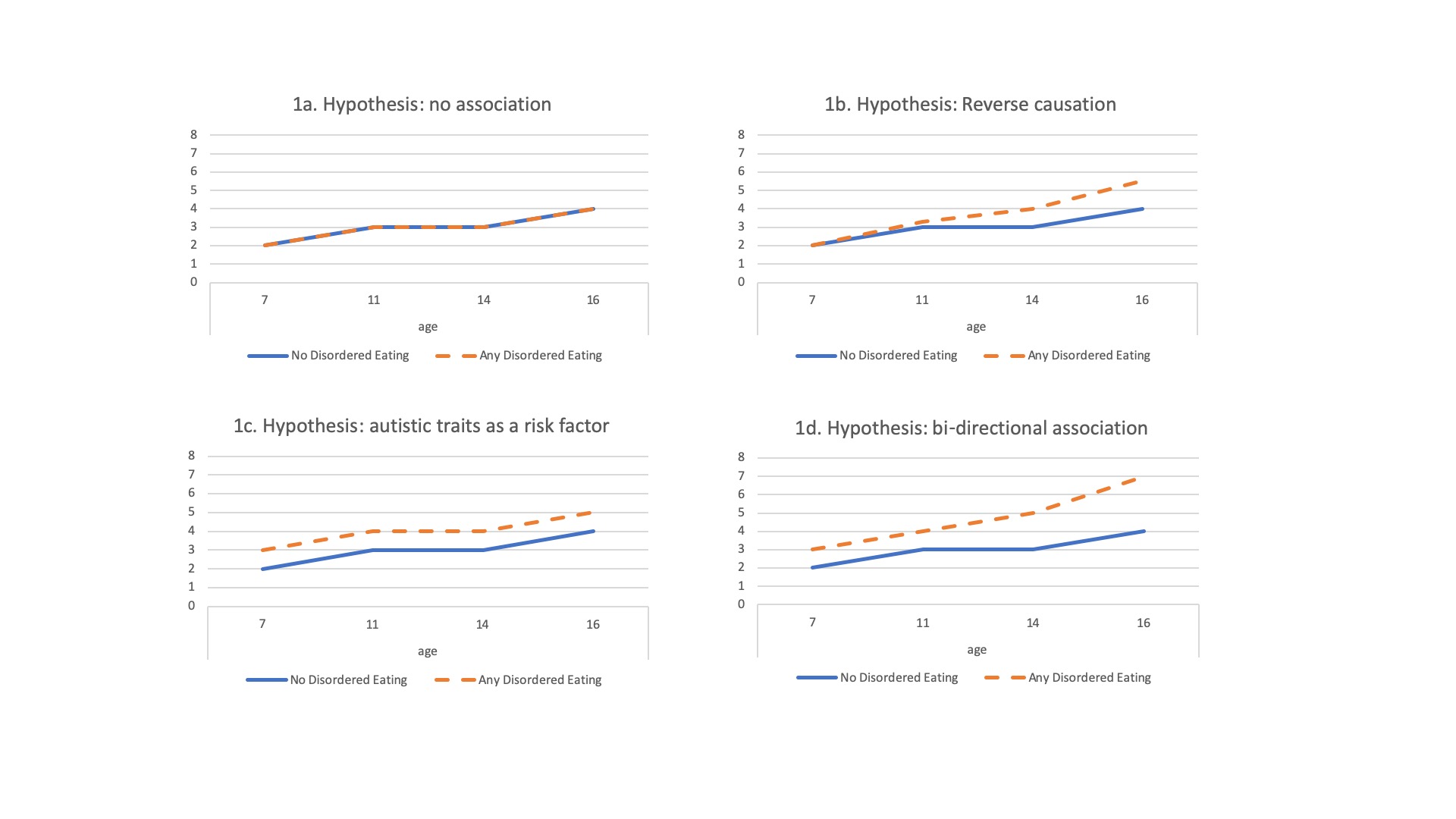


# Supplemental Figure 2: Definition of disordered eating behaviors in the disordered eating variable

**
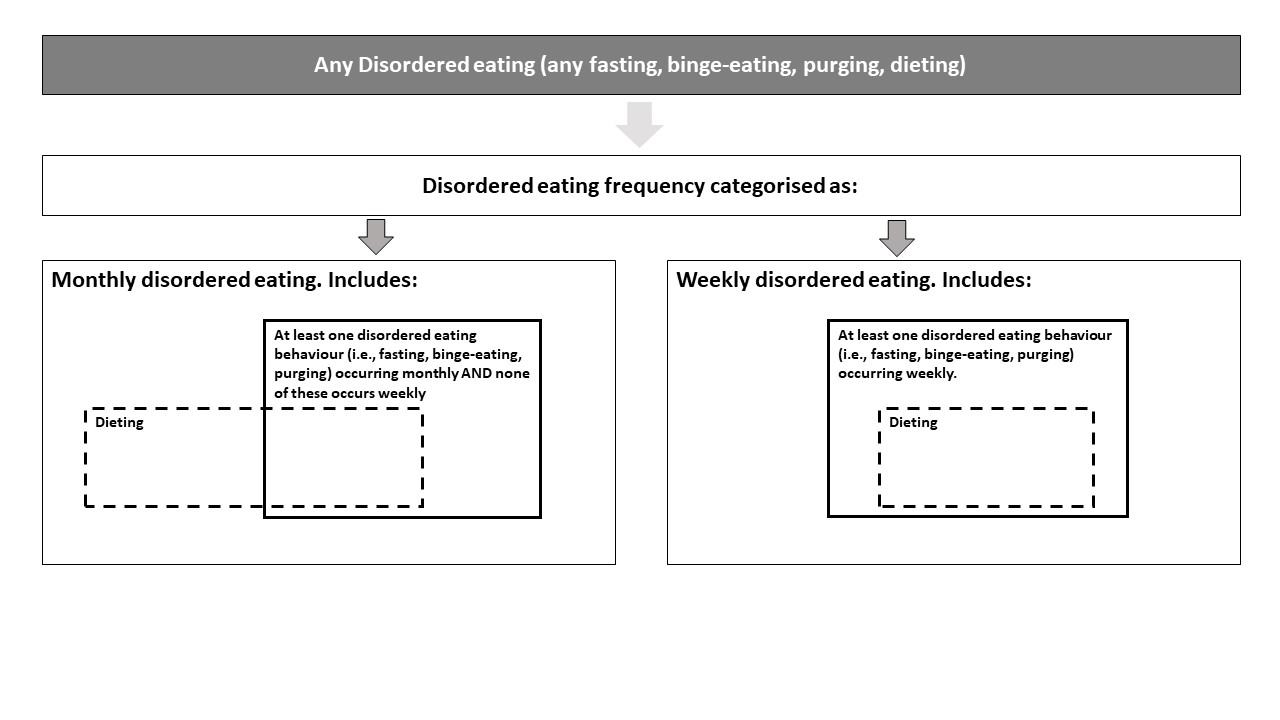
**

# Supplemental method 1: Choice of confounders

We adjusted our analyses for child’s sex as difficulties in social communication differ across childhood and adolescence between boys and girls^1^ and disordered eating behaviours are much more prevalent in girls.^2^ We additionally adjusted for child’s Body Mass Index (BMI) as a time-varying confounder BMI can drive changes in social communication abilities^3^ and high BMI is a risk factor for the development of disordered eating behaviours.^4^ Maternal age and education were included as proxies of socio-economic status, which is associated with autism spectrum disorders^5^ and disordered eating.^6^ Maternal pre-pregnancy BMI and depression have both been associated with disordered eating ^7,8^ and autism spectrum disorders. ^9,10^ Finally, we controlled our analyses for maternal history of eating disorders as there is evidence that children of mothers with a history of binge eating have

greater social communication difficulties ^11^ and that children of women with eating disorders are more likely to have an eating disorder. ^12^

# Supplemental method 2: Multiple imputation

We imputed 50 datasets using multiple imputation by chained equations and logistic, ordinal and multinomial logistic, and linear and truncated linear regression models depending on the nature of the variables to impute. As well as all variables used in the main analyses, we also included in our imputation models a number of auxiliary variables which we hypothesized would be associated with the exposure, disordered eating, and missingness. Auxiliary variables which we included in our multiple imputation models were: child’s weight and shape concerns and perceived pressures to lose weight at age 14; child’s depressive symptoms at age 12, 14, and 18 years; child’s total, verbal, and performance IQ at age 8; maternal weight and shape concerns; maternal marital status; child’s ethnicity and sexual orientation; child’s psychotic experiences at age 12 years, self-harm at age 16 years, and peer victimisation at age 8 and 11 years; child’s mental health difficulties (total Strength and Difficulties questionnaire score) at age 7, 9, 11 years; maternal smoking in pregnancy; and paternal and maternal occupational class.

# Supplemental Figure 3: Flowchart of study participation

**
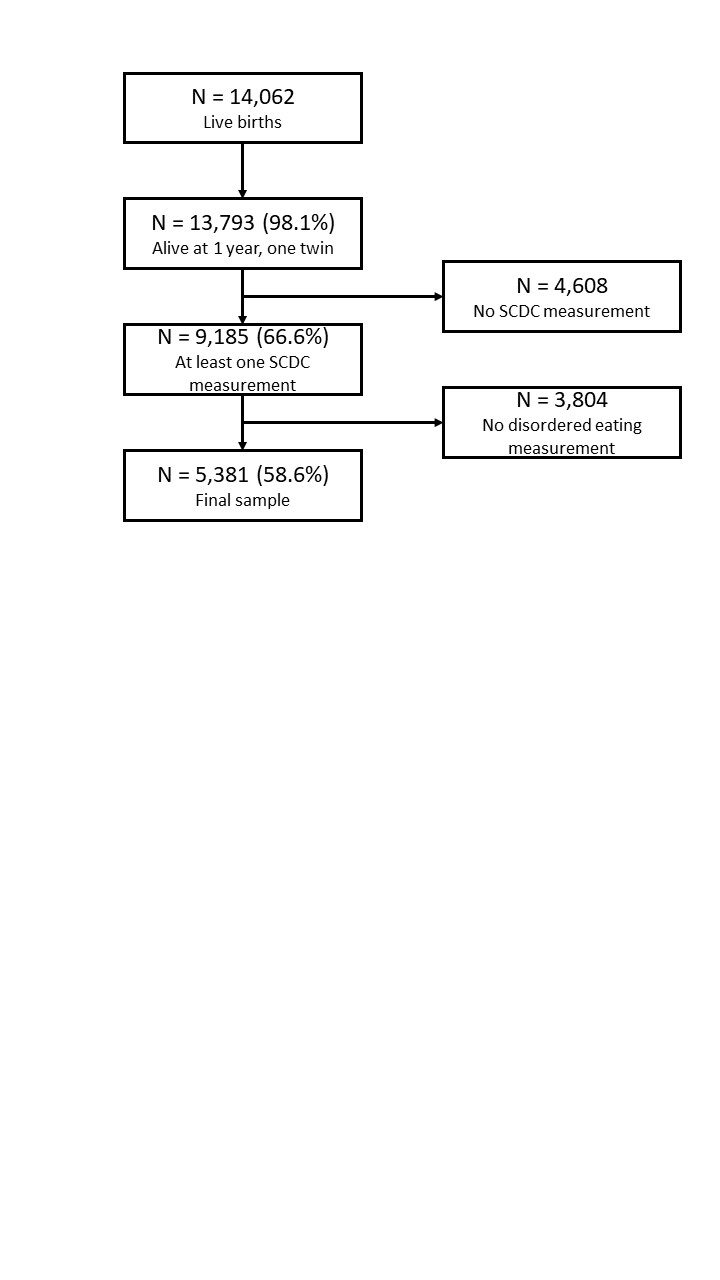
**

# Supplemental Table 1: Predictors of missing disordered eating data among girls with at least one SCDC measurements available

|  | **Missing outcome among those with at least one exposure measurement**  **(N = 9,185)**  **N (%)** |
| --- | --- |
| **Total with missing disordered eating data** | 3,804 (41.4%) |
|  | **OR (95% CI)** |
| **Child’s sex** |  |
| Male | Reference |
| Female | 0.55 (0.50, 0.59), p<0.0001 |
| **Maternal age** | 0.95 (0.94, 0.96), p<0.0001 |
| **Maternal depressive symptoms** | 1.04 (1.03, 1.05), p<0.0001 |
| **Maternal pre-pregnancy BMI** | 1.02 (1.01, 1.03), p=0.0002 |
| **Maternal education** |  |
| Compulsory | Reference |
| Non-compulsory | 0.54 (0.50, 0.59), p<0.0001 |
| **Paternal Social class** |  |
| Manual | Reference |
| Non-manual | 0.63 (0.58, 0.69), p<0.0001 |
| **Autistic social traits age 7 years** | 1.04 (1.03, 1.06), p<0.0001 |
| **Autistic social traits age 11 years** | 1.07 (1.06, 1.08), p<0.0001 |
| **Child’s BMI at age 7 years** | 1.03 (0.98, 1.09), p=0.2228 |
| **Child’s BMI at age 11 years** | 1.06 (1.01, 1.12), p=0.0206 |

# Supplemental Table 2: Results of the unconditional model (model 1) only modelling trajectories of autistic social traits between age 7 and 16 years among participants by age and age squared values. Sample based on participants with complete disordered eating data, at least one SCDC measurement and imputed confounders. (N = 5,381)

|  | **Model 1 relative risk**  **(95% CI), p-value** |
| --- | --- |
| **Age** | 1.027 (1.019, 1.035), p<0.0001 |
| **Age^2^** | 1.013 (1.011, 1.015), p<0.0001 |

# Supplemental Table 3: Proportion of children with disordered eating behaviours in the main analytical sample , n=5,381

|  | **Females** | | | **Males** | | |
| --- | --- | --- | --- | --- | --- | --- |
|  | **Any**  **n(%)** | **Monthly**  **n(%)** | **Weekly**  **n(%)** | **Any**  **n(%)** | **Monthly**  **n(%)** | **Weekly**  **n(%)** |
| **Fasting** | 130 (4.3%) | 52 (1.7%) | 78 (2.6%) | 20 (0.8%) | 7 (0.3%) | 13 (0.5%) |
| **Dieting** | 178 (5.8%) | - | - | 43 (1.7%) | - | - |
| **Binge eating** | 83 (2.7%) | 44 (1.5%) | 39 (1.3%) | 31 (1.2%) | 12 (0.5%) | 19 (0.8%) |
| **Purging** | 27 (0.9%) | 8 (0.3%) | 19 (0.6%) | 7 (0.3%) | * | * |

***** cell count in either of these cells is <5 hence cannot be reported as per ALSPAC rules

# Supplemental Table 4 Results of the unconditional model (model 1) only modelling trajectories of autistic social traits between age 7 and 16 years among participants by age and age squared values in boys and girls, separately. Sample based on participants with complete disordered eating data, at least one SCDC measurement and imputed confounders. (Girls, n= 2,971, boys, n= 2,410)

|  | **Model 1 relative risk**  **(95% CI), p-value** |
| --- | --- |
| **Girls** |  |
| *Age* | 1.043 (1.034, 1.053), p<0.0001 |
| *Age^2^* | 1.014 (1.011, 1.018), p<0.0001 |
| **Boys** |  |
| *Age* | 1.008 (0.998, 1.018), p=0.1013 |
| *Age^2^* | 1.010 (1.007, 1.014), p<0.0001 |

# Supplemental Figure 4: Trajectories of autistic social traits by child’s sex and presence of disordered eating behaviours at age 14 years, derived from Model 3 predictions. Sample based on participants with complete disordered eating data, at least 1 SCDC measurements and imputed confounders. (N girls = 2,464, N boys = 1,890)

# Supplemental Figure 5: Trajectories of autistic social traits in girls by frequency of disordered eating behaviours at age 14 years, derived from Model 3 predictions. Sample based on participants with complete disordered eating data, at least 1 SCDC measurements and imputed confounders. (N = 2,464)

# Supplemental Table 5: Multilevel negative binomial regression modelling trajectories of social communication difficulties between age 7 and 16 years among participants with disordered eating at age 14 years stratified by participants’ sex. Sample based on participants with complete disordered eating data, at least one SCDC measurement and imputed confounders. (Girls, n= 2,971, boys, n= 2,410)

| **Autistic social traits** | | | | |
| --- | --- | --- | --- | --- |
|  | **Univariable model 2**  **Relative Risk^a^ (95% CI)** | **Adjusted model 3**  **relative risk (95% CI)** | **Adjusted model 4**  **relative risk (95% CI)** | **Adjusted model 5**  **relative risk (95% CI)** |
| **Any disordered eating behaviours (girls, n= 2,971)** |  |  |  |  |
| *No* | Reference | Reference | Reference | Reference |
| *Yes* | 1.27 (1.17, 1.38), p<0.0001 | 1.21 (1.12, 1.32), p<0.0001 | 1.21 (1.12, 1.32), p<0.0001 | 1.26 (1.09, 1.43), p=0.0009 |
| ***Any DEB*time*** |  | - | 1.01 (0.98, 1.03), p=0.52 | 1.01 (0.98, 1.03), p=0.76 |
| ***Any DEB*time^2^*** |  | - | - | 0.99 (0.99, 1.01), p=0.50 |
|  | **Univariable model 2**  **Relative Risk^a^ (95% CI)** | **Adjusted model 3**  **relative risk (95% CI)** | **Adjusted model 4**  **relative risk (95% CI)** | **Adjusted model 5**  **relative risk (95% CI)** |
| **Any disordered eating behaviours (boys, n= 2,410)** |  |  |  |  |
| *No* | Reference | Reference | Reference | Reference |
| *Yes* | 1.37 (1.18, 1.60), p<0.0001 | 1.30 (1.12, 1.52), p=0.0008 | 1.30 (1.12, 1.51), p=0.0008 | 1.22 (0.95, 1.55), p=0.11 |
| ***Any DEB *time*** |  | - | 1.02 (0.98, 1.06), p=0.41 | 1.02 (0.98, 1.07), p=0.32 |
| ***Any DEB *time^2^*** |  | - | - | 0.99 (0.99, 1.02),p=0.56 |

Model 2 = model 1 + disordered eating variable.

Model 3= model 2 + child’s sex, maternal age, maternal pre-pregnancy BMI, maternal depressive symptoms in pregnancy, maternal history of eating disorders, and maternal education. Child BMI was adjusted for as a time-varying confounder.

Model 4= model 3 + eating disorder variable*time interaction

Model 5 = model 4 + eating disorder variable* time^2^ interaction

**^a^** Relative Risk is derived from exponentiating the coefficients of multilevel negative binomial regressions, modelling the logs of SCDC scores in adolescents with disordered eating (i.e., any, monthly, or weekly) compared to those with no disordered eating, holding confounder variables in the model constant.

Abbreviations: BMI=Body Mass Index, CI=confidence interval, DEB=Disordered Eating Behaviour, SCDC=Social Communication and Development Checklist.

# Supplemental Table 6: Multilevel negative binomial regression modelling trajectories of social communication difficulties between age 7 and 16 years among girls with monthly and weekly disordered eating at age 14 years. Sample based on participants with complete disordered eating data, at least one SCDC measurement and imputed confounders. (n = 2,971)

| **Autistic social traits** | | | | |
| --- | --- | --- | --- | --- |
|  | **Univariable model 2**  **Relative Risk^a^ (95% CI)** | **Adjusted model 3**  **relative risk (95% CI)** | **Adjusted model 4**  **relative risk (95% CI)** | **Adjusted model 5**  **relative risk (95% CI)** |
| **Severity of disordered eating behaviours ^a^** |  |  |  |  |
| *None* | Reference | Reference | Reference | Reference |
| *Behaviours occurring monthly* | 1.11 (1.01, 1.23), p=0.036 | 1.07 (0.97, 1.19), p=0.17 | 1.07 (0.97, 1.19), p=0.17 | 1.06 (0.90.1.26), p=0.47 |
| *Behaviours occurring weekly* | 1.56 (1.37, 1.78), p<0.0001 | 1.48 (1.29, 1.68), p<0.0001 | 1.47 (1.29, 1.68), p<0.0001 | 1.62 (1.31, 1.82), p<0.0001 |
| ***Monthly DEB *time*** |  | - | 1.01 (0.98, 1.04), p=0.54 | 1.01 (0.98, 1.04), p=0.54 |
| ***Weekly DEB *time*** |  | - | 1.01 (0.97, 1.04), p=0.76 | 0.99 (0.95, 1.04), p=0.87 |
| ***Monthly DEB *time^2^*** |  | - | - | 1.01 (0.99, 1.01), p=0.90 |
| ***Weekly DEB *time^2^*** |  | - | - | 0.99 (0.98, 1.01), p=0.26 |

Model 2 = model 1 + disordered eating variable.

Model 3= model 2 + child’s sex, maternal age, maternal pre-pregnancy BMI, maternal depressive symptoms in pregnancy, maternal history of eating disorders, and maternal education. Child BMI was adjusted for as a time-varying confounder.

Model 4= model 3 + eating disorder variable*time interaction

Model 5 = model 4 + eating disorder variable* time^2^ interaction

**^a^** Relative Risk is derived from exponentiating the coefficients of multilevel negative binomial regressions, modelling the logs of SCDC scores in adolescents with disordered eating (i.e., any, monthly, or weekly) compared to those with no disordered eating, holding confounder variables in the model constant.

Abbreviations: BMI=Body Mass Index, CI=confidence interval, DEB=Disordered Eating Behaviour, SCDC=Social Communication and Development Checklist.

# Supplemental method 3: Sensitivity analyses

Analyses based on adolescents with at least two exposure measurements available and imputed confounders and disordered eating data (n= 5,408) yielded comparable results, despite a more conservative approach to sample definition (**Supplemental Table 7**). Results were also comparable when we ran our models on all adolescents with at least one exposure measurement and imputed confounders and disordered eating data (n=9,185, **Supplemental Table 8**)

Similarly, analyses using multilevel mixed linear regressions, were comparable in size, direction, and strength of the association (**Supplemental Table 9**), though here we found some evidence for a linear interaction between time and disordered eating.

# Supplemental Table 7: Multilevel negative binomial regression modelling trajectories of social communication difficulties between age 7 and 16 years among participants with disordered eating at age 14 years. Sample based on adolescents with complete disordered eating data, at least two SCDC measurements, and imputed confounders (n=5,048).

| **Autistic social traits** | | | | |
| --- | --- | --- | --- | --- |
|  | **Univariable model 2**  **Relative Risk^a^ (95% CI)** | **Adjusted model 3**  **relative risk (95% CI)** | **Adjusted model 4**  **relative risk (95% CI)** | **Adjusted model 5**  **relative risk (95% CI)** |
| **Any disordered eating behaviors** |  |  |  |  |
| *No* | Reference | Reference | Reference | Reference |
| *Yes* | 1.23 (1.14, 1.32), p<0.0001 | 1.22 (0.13, 1.31), p<0.0001 | 1.21 (0.12, 1.31), p<0.0001 | 1.21 (1.06, 1.37)), p=0.004 |
| ***Any ED*time*** |  | - | 1.02 (0.99, 1.04), p=0.11 | 1.02 (0.99, 1.04), p=0.14 |
| ***Any ED*time^2^*** |  | - | - | 1.00 (0.99, 1.01), p=0.91 |
|  | **Univariable model 2**  **Relative Risk^a^ (95% CI)** | **Adjusted model 3**  **relative risk (95% CI)** | **Adjusted model 4**  **relative risk (95% CI)** | **Adjusted model 5**  **relative risk (95% CI)** |
| **Severity of disordered eating behaviors ^a^** |  |  |  |  |
| *None* | Reference | Reference | Reference | Reference |
| *Behaviors occurring monthly* | 1.13 (1.03, 1.23), p=0.009 | 1.12 (1.01, 1.22), p=0.02 | 1.11 (1.02, 1.22), p=0.02 | 1.08 (0.93, 1.26), p=0.31 |
| *Behaviors occurring weekly* | 1.42 (1.26, 1.61), p<0.0001 | 1.40 (1.25, 1.60), p<0.0001 | 1.41 (1.24, 1.59), p<0.0001 | 1.45 (1.19, 1.78), p=0.0003 |
| ***Monthly DEB*time*** |  | - | 1.02 (0.99, 1.05), p=0.16 | 1.02 (0.99, 1.05), p=0.14 |
| ***Weekly DEB *time*** |  | - | 1.02 (0.98, 1.05), p=0.40 | 1.01 (0.97, 1.05), p=0.54 |
| ***Monthly DEB *time^2^*** |  | - | - | 1.00 (0.99, 1.01), p=0.63 |
| ***Weekly DEB *time^2^*** |  | - | - | 1.00 (0.98, 1.01), p=0.71 |

Model 2 = model 1 + disordered eating variable.

Model 3= model 2 + child’s sex, maternal age, maternal pre-pregnancy BMI, maternal depressive symptoms in pregnancy, maternal history of eating disorders, and maternal education. Child BMI was adjusted for as a time-varying confounder.

Model 4= model 3 + eating disorder variable*time interaction

Model 5 = model 4 + eating disorder variable* time^2^ interaction

**^a^** Relative Risk is derived from exponentiating the coefficients of multilevel negative binomial regressions, modelling the logs of SCDC scores in adolescents with disordered eating (i.e., any, monthly, or weekly) compared to those with no disordered eating, holding confounder variables in the model constant.

Abbreviations: BMI=Body Mass Index, CI=confidence interval, DEB=Disordered Eating Behaviour, SCDC=Social Communication and Development Checklist.

# Supplemental Table 8: Multilevel negative binomial regression modelling trajectories of social communication difficulties between age 7 and 16 years among participants with disordered eating at age 14 years. Sample based on adolescents with at least one SCDC measurements, and imputed disordered eating and confounders (n=9,185).

| **Autistic social traits** | | | | |
| --- | --- | --- | --- | --- |
|  | **Univariable model 2**  **Relative Risk^a^ (95% CI)** | **Adjusted model 3**  **relative risk (95% CI)** | **Adjusted model 4**  **relative risk (95% CI)** | **Adjusted model 5**  **relative risk (95% CI)** |
| **Any disordered eating behaviors** |  |  |  |  |
| *No* | Reference | Reference | Reference | Reference |
| *Yes* | 1.25 (1.13, 1.38), p<0.0001 | 1.23 (1.12, 1.35), p<0.0001 | 1.23 (1.12, 1.35), p<0.0001 | 1.23 (1.07, 1.41), p=0.0036 |
| ***Any ED*time*** |  | - | 1.01 (0.99, 1.03), p=0.1473 | 1.01 (0.99, 1.03), p=0.1867 |
| ***Any ED*time^2^*** |  | - | - | 0.99 (0.99, 1.00), p=0.9781 |
|  | **Univariable model 2**  **Relative Risk^a^ (95% CI)** | **Adjusted model 3**  **relative risk (95% CI)** | **Adjusted model 4**  **relative risk (95% CI)** | **Adjusted model 5**  **relative risk (95% CI)** |
| **Severity of disordered eating behaviors ^a^** |  |  |  |  |
| *None* | Reference | Reference | Reference | Reference |
| *Behaviors occurring monthly* | 1.17 (1.04, 1.31), p=0.008 | 1.15 (1.03, 1.28), p=0.0127 | 1.15 (1.03, 1.28), p=0.0135 | 1.14 (0.97, 1.32), p=0.1059 |
| *Behaviors occurring weekly* | 1.40 ( | 1.36 (1.18, 1.57), p<0.0001 | 1.36 (1.18, 1.57), p<0.0001 | 1.41 (1.15, 1.70), p=0.0010 |
| ***Monthly*time*** |  |  | 1.01 (0.99, 1.04), p=0.2035 | 1.01 (0.99, 1.04), p=0.1993 |
| ***Weekly*time*** |  |  | 1.01 (0.98, 1.04), p=0.3651 | 1.01 (0.98, 1.04), p=0.5096 |
| ***Monthly*time^2^*** |  |  |  | 1.01 (0.99, 1.01), p=0.7700 |
| ***Weekly*time^2^*** |  |  |  | 0.99 (0.99, 1.01), p=0.7028 |

Model 2 = model 1 + disordered eating variable.

Model 3= model 2 + child’s sex, maternal age, maternal pre-pregnancy BMI, maternal depressive symptoms in pregnancy, maternal history of eating disorders, and maternal education. Child BMI was adjusted for as a time-varying confounder.

Model 4= model 3 + eating disorder variable*time interaction

Model 5 = model 4 + eating disorder variable* time^2^ interaction

**^a^** Relative Risk is derived from exponentiating the coefficients of multilevel negative binomial regressions, modelling the logs of SCDC scores in adolescents with disordered eating (i.e., any, monthly, or weekly) compared to those with no disordered eating, holding confounder variables in the model constant.

Abbreviations: BMI=Body Mass Index, CI=confidence interval, DEB=Disordered Eating Behaviour, SCDC=Social Communication and Development Checklist.

# Supplemental Table 9: Multilevel linear mixed regression modelling trajectories of social communication difficulties between age 7 and 16 years among participants with disordered eating at age 14 years. Sample based on adolescents with at least two SCDC measurements, complete disordered eating and imputed confounders. (n=5,831)

| **Autistic social traits** | | | | |
| --- | --- | --- | --- | --- |
|  | **Univariable model 2**  **Relative Risk^a^ (95% CI)** | **Adjusted model 3**  **relative risk (95% CI)** | **Adjusted model 4**  **relative risk (95% CI)** | **Adjusted model 5**  **relative risk (95% CI)** |
| **Any disordered eating behaviors** |  |  |  |  |
| *No* | Reference | Reference | Reference | Reference |
| *Yes* | 0.61 (0.32, 0.89), p<0.0001 | 0.59 (0.29, 0.89), p<0.0001 | 0.62 (0.31, 0.93), p<0.0001 | 0.53 (0.17, 0.89), p=0.0040 |
| ***Any ED*time*** |  |  | 0.06 (0.01, 0.11), p=0.0200 | 0.07 (0.02, 012), p=0.0086 |
| ***Any ED*time^2^*** |  |  | - | 0.01 (-0.01, 0.02), p=0.2799 |
|  | **Univariable model 2**  **Relative Risk^a^ (95% CI)** | **Adjusted model 3**  **relative risk (95% CI)** | **Adjusted model 4**  **relative risk (95% CI)** | **Adjusted model 5**  **relative risk (95% CI)** |
| **Severity of disordered eating behaviors ^a^** |  |  |  |  |
| *None* | Reference | Reference | Reference | Reference |
| *Behaviors occurring monthly* | 0.33 (-0.01, 0.67) p=0.06 | 0.34 (-0.01, 0.69), p=0.059 | 0.37 (0.01, 0.73), p=0.046 | 0.24 (-0.18, 0.65), p=0.264 |
| *Behaviors occurring weekly* | 1.11 (0.64, 1.59), p<0.0001 | 1.05 (0.53, 1.57), p<0.0001 | 1.09 (0.56, 1.62), p<0.0001 | 1.06 (0.45, 1.68), p=0.001 |
| ***Monthly*time*** |  |  | 0.05 (-0.01, 0.11), p=0.093 | 0.06 (-0.01, 0.13), p=0.057 |
| ***Weekly*time*** |  |  | 0.08 (-0.01, 0.18), p=0.095 | 0.08 (-0.03, 0.20), p=0.136 |
| ***Monthly*time^2^*** |  |  | - | 0.01 (-0.01, 0.030), p=0.262 |
| ***Weekly*time^2^*** |  |  | - | 0.01 (-0.03, 0.03), p=0.866 |

Model 2 = model 1 + disordered eating variable.

Model 3= model 2 + child’s sex, maternal age, maternal pre-pregnancy BMI, maternal depressive symptoms in pregnancy, maternal history of eating disorders, and maternal education. Child BMI was adjusted for as a time-varying confounder.

Model 4= model 3 + eating disorder variable*time interaction

Model 5 = model 4 + eating disorder variable* time^2^ interaction

**^a^** Relative Risk is derived from exponentiating the coefficients of multilevel negative binomial regressions, modelling the logs of SCDC scores in adolescents with disordered eating (i.e., any, monthly, or weekly) compared to those with no disordered eating, holding confounder variables in the model constant.

Abbreviations: BMI=Body Mass Index, CI=confidence interval, DEB=Disordered Eating Behaviour, SCDC=Social Communication and Development Checklist.

# References

1 Mandy W, Pellicano L, St Pourcain B, Skuse D, Heron J. The development of autistic social traits across childhood and adolescence in males and females. *J Child Psychol Psychiatry* 2018; published online April 19. DOI:10.1111/jcpp.12913.

2 Micali N, Horton NJ, Crosby RD, *et al.* Eating disorder behaviours amongst adolescents: investigating classification, persistence and prospective associations with adverse outcomes using latent class models. *Eur Child Adolesc Psychiatry* 2017; **26**: 231–40.

3 Hiller R, Pellicano L. Autism and anorexia: A cautionary note. *Psychologist* 2013; **26**: 780.

4 Reed ZE, Micali N, Bulik CM, Smith GD, Wade KH. Assessing the causal role of adiposity on disordered eating in childhood, adolescence, and adulthood: A Mendelian randomization analysis. *Am J Clin Nutr* 2017; **106**: 764–72.

5 Russell G, Rodgers LR, Ukoumunne OC, Ford T. Prevalence of parent-reported ASD and ADHD in the UK: Findings from the millennium cohort study. *J Autism Dev Disord* 2014. DOI:10.1007/s10803-013-1849-0.

6 Larsen PS, Strandberg-Larsen K, Olsen EM, Micali N, Nybo Andersen A-M. Parental characteristics in association with disordered eating in 11- to 12-year-olds: A study within the Danish National Birth Cohort. *Eur Eat Disord Rev* 2018; published online April 27. DOI:10.1002/erv.2599.

7 Micali N, Daniel RM, Ploubidis GB, De Stavola BL. Maternal Prepregnancy Weight Status and Adolescent Eating Disorder Behaviors: A Longitudinal Study of Risk Pathways. *Epidemiology* 2018; **29**: 579–89.

8 Bould H, Koupil I, Dalman C, DeStavola B, Lewis G, Magnusson C. Parental mental illness and eating disorders in offspring. *Int J Eat Disord* 2015; **48**: 383–91.

9 Wang Y, Tang S, Xu S, Weng S, Liu Z. Maternal body mass index and risk of autism spectrum disorders in offspring: A meta-analysis. *Sci Rep* 2016. DOI:10.1038/srep34248.

10 Rai D, Lee BK, Dalman C, Golding J, Lewis G, Magnusson C. Parental depression, maternal antidepressant use during pregnancy, and risk of autism spectrum disorders: Population based case-control study. *BMJ* 2013. DOI:10.1136/bmj.f2059.

11 Kothari R, Barona M, Treasure J, Micali N. Social cognition in children at familial high-risk of developing an eating disorder. *Front Behav Neurosci* 2015; **9**: 1–17.

12 Bould H, Sovio U, Koupil I, *et al.* Do eating disorders in parents predict eating disorders in children? Evidence from a Swedish cohort. *Acta Psychiatr Scand* 2015; **132**: 51–9.
